# Supplementary figures and images for: Quantitative real-time PCR analysis of bacterial biomarkers enable fast and accurate monitoring in inflammatory bowel disease
Source: PeerJ. 2022 Oct 18;10:e14217. doi: 10.7717/peerj.14217 (PMC9586115; doi:10.7717/peerj.14217)

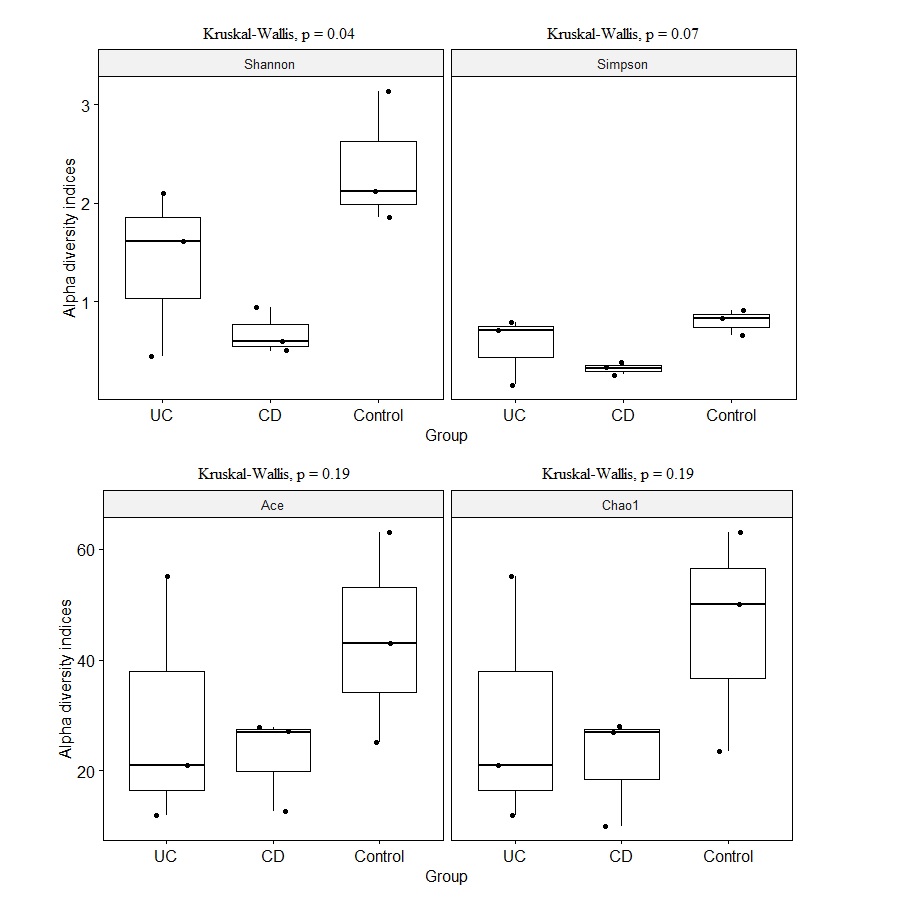

Supplement: Supplemental Information 1 [file peerj-10-14217-s001.jpg]

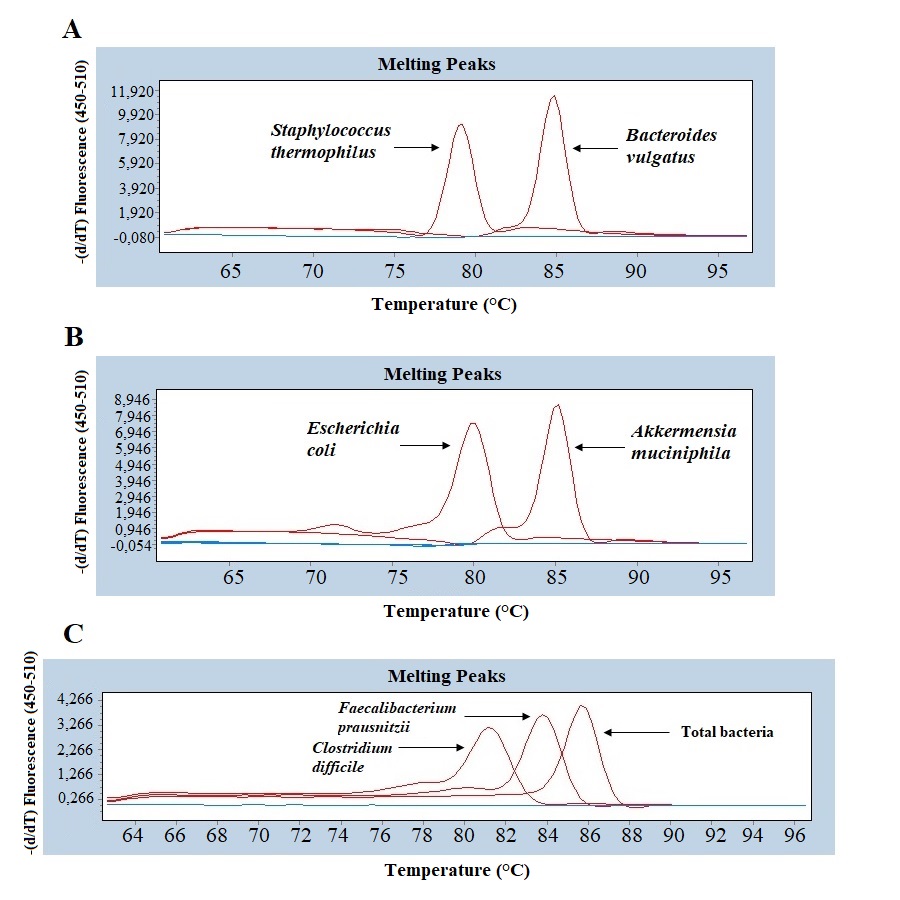

Supplement: Supplemental Information 2 [file peerj-10-14217-s002.jpg]
