# Supplementary material for: Quantitative real-time PCR analysis of bacterial biomarkers enable fast and accurate monitoring in inflammatory bowel disease
Source: PeerJ. 2022 Oct 18;10:e14217. doi: 10.7717/peerj.14217 (PMC9586115; doi:10.7717/peerj.14217)
Supplement: Supplemental Information 3 [file peerj-10-14217-s003.docx]

**Supplemental Table 1.** Primers targeting IBD related genes and their variants

|  |  |  | **Primers** | | |
| --- | --- | --- | --- | --- | --- |
| **Gene** | **SNP** |  | **Direction** |  | **Primer sequence** |
| *NOD2* |  |  |  |  |  |
|  | rs2066844 |  | Forward |  | CATGTAGCCCAACTTTCTCAAAC |
|  |  |  | Reverse |  | AGAGAGTTTGGCATGGGTAAG |
|  | rs2066845 |  | Forward |  | CATGTGCTGGCTCTCTTTCT |
|  |  |  | Reverse |  | TGACTGGCAACTCACTCTAAAC |
|  | rs2066847 |  | Forward |  | TTTCTCTTGGCTTCCTGGTG |
|  |  |  | Reverse |  | TTGAGGTGCCCAACATTCA |
|  |  |  |  |  |  |
| *ATG16L1* |  |  |  |  |  |
|  | rs2241880 |  | Forward |  | TTAAGGGTTAGGGCTGGGTA |
|  |  |  | Reverse |  | CGAGTGTCCTCTCTTGAAATCC |
|  |  |  |  |  |  |
| *IL23R* |  |  |  |  |  |
|  | rs11209026 |  | Forward |  | ACTGATGGTACTGAGCCTTTG |
|  |  |  | Reverse |  | CCTGCTCCTAACCTGTGTAATC |
