# Supplementary material for: Quantitative real-time PCR analysis of bacterial biomarkers enable fast and accurate monitoring in inflammatory bowel disease
Source: PeerJ. 2022 Oct 18;10:e14217. doi: 10.7717/peerj.14217 (PMC9586115; doi:10.7717/peerj.14217)
Supplement: Supplemental Information 4 [file peerj-10-14217-s004.docx]

**Supplemental Table 2.** Control, patient group, and disease characteristics for samples included in 16S rRNA gene NGS analysis

|  | **Crohn’s disease (%) N=3** | **Ulcerative colitis (%) N=3** | **Control (%) N=3** | |
| --- | --- | --- | --- | --- |
| **Median Age, years (25%,75%)** | 55 (52,62) | 39 (37,43) | 24 (22,24) |  |
| **Sex (%)** |  |  |  |  |
| Female | 1 (33) | 2 (67) | 2 (67) |  |
| Male | 2 (67) | 1 (33) | 1 (33) |  |
|  |  |  | - |  |
| **Median disease duration,**  **years (25%,75%)** | 9 (7,10) | 10 (5,13) | - |  |
| **Smoking History** |  |  |  |  |
| Yes (%) | 1 (33) | 0 (0) | 1 (33) |  |
| **Disease localization (%)** |  |  |  |  |
| Ileal | 0 (0) | - | - |  |
| Colonic | 0 (0) | - | - |  |
| Ileocolonic | 1 (33) | - | - |  |
| Surgery | 2 (67) | - | - |  |
| Penetrating perianal disease | 1 (33) | - | - |  |
| Distal colitis | - | 1 (33) | - |  |
| Left colitis | - | 1 (33) | - |  |
| Pancolitis | - | 1 (33) | - |  |
|  |  |  |  |  |
| **Treatment (%)** |  |  | - |  |
| Biologics | 3 (100) | 2 (67) | - |  |
| Non-biologics | 0 (0) | 1 (33) | - |  |
|  |  |  |  |  |
| **Median Mayo Score (25%,75%)** | - | 6 (5,6) | - |  |
| **Harvey-Bradshaw Index**  **(25%,75%)** | 7 (7,8) | - | - |  |
|  |  |  |  |  |
| **C-reactive protein (CRP mg/dL)** |  |  |  |  |
| <5 (%) | 1 (33) | 3 (100) | - |  |
| >5 (%) | 2 (67) | 0 (0) | - |  |

Note: CRP level at fecal sample collection time indicating severity of disease activity
