# Supplementary material for: Quantitative real-time PCR analysis of bacterial biomarkers enable fast and accurate monitoring in inflammatory bowel disease
Source: PeerJ. 2022 Oct 18;10:e14217. doi: 10.7717/peerj.14217 (PMC9586115; doi:10.7717/peerj.14217)
Supplement: Supplemental Information 5 [file peerj-10-14217-s005.docx]

**Supplemental Table 3.** Distribution of genotypic frequencies of *ATG16L1*, *IL23R* and *NOD2* variants among CD and UC patients

| **Gene / SNP (alleles)** | **Genotype** | **CD (%)** | **UC (%)** | **p-value** |
| --- | --- | --- | --- | --- |
| ***ATG16L1* /**  **rs2241880 (A/G)** | AA ^Wild type^ | 3 (25) | 1 (14.2) | 0.69 |
|  | AG ^Heterozygote^ | 3 (25) | 3 (42.9) |  |
|  | GG ^Mutation^ | 6 (50) | 3 (42.9) |  |
| ***IL23R* /**  **rs11209026 (G/A)** | GG ^Wild type^ | 10 (83.3) | 7 (100) | - |
|  | GA ^Heterozygote^ | 2 (16.7) | 0 |  |
|  | AA ^Mutation^ | 0 | 0 |  |
| ***NOD2* /**  **rs2066844 (C/T)** | CC ^Wild type^ | 12 (100) | 7 (100) | - |
|  | CT ^Heterozygote^ | 0 | 0 |  |
|  | TT ^Mutation^ | 0 | 0 |  |
| ***NOD2* /**  **rs2066845 (G/C)** | GG ^Wild type^ | 12 (100) | 7 (100) | - |
|  | GC ^Heterozygote^ | 0 | 0 |  |
|  | CC ^Mutation^ | 0 | 0 |  |
| ***NOD2* /**  **rs2066847 ()** | Wild type | 12 (100) | 7 (100) | - |
|  | Heterozygote | 0 | 0 |  |
|  | Mutation | 0 | 0 |  |
