# Supplementary material for: Quantitative real-time PCR analysis of bacterial biomarkers enable fast and accurate monitoring in inflammatory bowel disease
Source: PeerJ. 2022 Oct 18;10:e14217. doi: 10.7717/peerj.14217 (PMC9586115; doi:10.7717/peerj.14217)
Supplement: Supplemental Information 6 [file peerj-10-14217-s006.docx]

**Supplemental Table 4.** Bacterial species unique to and shared between Ulcerative Colitis (UC), Crohn disease (CD), and Control groups

| **Group** | **Bacterial Species** |
| --- | --- |
| Only found in UC | \| *Actinomyces odontolyticus* \| \| --- \| \| *Actinomyces turicensis* \| \| *Aeromonas caviae* \| \| *Aggregatibacter aphrophilus* \| \| *Anaerococcus vaginalis* \| \| *Bacteroides finegoldii* \| \| *Bifidobacterium pseudolongum* \| \| *Citrobacter freundii* \| \| *Clostridium butyricum* \| \| *Clostridium chromiireducens* \| \| *Clostridium clostridioforme* \| \| *Clostridium innocuum* \| \| *Clostridium paraputrificum* \| \| *Clostridium polysaccharolyticum* \| \| *Clostridium tertium* \| \| *Comamonas kerstersii* \| \| *Cuneatibacter caecimuris* \| \| *Dialister invisus* \| \| *Dorea formicigenerans* \| \| *Eggerthella lenta* \| \| *Eikenella corrodens* \| \| *Eisenbergiella tayi* \| \| *Enterococcus canis* \| \| *Enterococcus casseliflavus* \| \| *Enterococcus durans* \| \| *Enterococcus faecalis* \| \| *Enterococcus faecium* \| \| *Enterococcus saigonensis* \| \| *Erysipelatoclostridium ramosum* \| \| *Eubacterium ramulus* \| \| *Faecalicatena orotica* \| \| *Fusobacterium gastrosuis* \| \| *Fusobacterium periodonticum* \| \| *Fusobacterium simiae* \| \| *Fusobacterium varium* \| \| *Haemophilus aegyptius* \| \| *Haemophilus haemolyticus* \| \| *Haemophilus massiliensis* \| \| *Haemophilus parainfluenzae* \| \| *Haemophilus paraphrohaemolyticus* \| \| *Haemophilus pittmaniae* \| \| *Holdemanella biformis* \| \| *Hungatella effluvii* \| \| *Intestinibacter bartlettii* \| \| *Klebsiella pneumoniae* \| \| *Klebsiella variicola* \| \| *Lactobacillus brantae* \| \| *Lactobacillus fermentum* \| \| *Lactobacillus gallinarum* \| \| *Lactobacillus parabuchneri* \| \| *Lactobacillus porcinae* \| \| *Lactobacillus rhamnosus* \| \| *Lactobacillus salivarius* \| \| *Leptotrichia trevisanii* \| \| *Leptotrichia wadei* \| \| *Megasphaera micronuciformis* \| \| *Muricomes intestini* \| \| *Parvimonas micra* \| \| *Pediococcus stilesii* \| \| *Peptoniphilus duerdenii* \| \| *Peptoniphilus grossensis* \| \| *Peptoniphilus koenoeneniae* \| \| *Peptostreptococcus anaerobius* \| \| *Peptostreptococcus stomatis* \| \| *Prevotella buccae* \| \| *Prevotella buccalis* \| \| *Prevotella denticola* \| \| *Prevotella melaninogenica* \| \| *Prevotella oris* \| \| *Prevotella shahii* \| \| *Prevotella stercorea* \| \| *Rothia mucilaginosa* \| \| *Shigella sonnei* \| \| *Solobacterium moorei* \| \| *Streptococcus agalactiae* \| \| *Streptococcus alactolyticus* \| \| *Streptococcus gordonii* \| \| *Streptococcus hongkongensis* \| \| *Streptococcus macedonicus* \| \| *Streptococcus massiliensis* \| \| *Streptococcus mitis* \| \| *Streptococcus porcorum* \| \| *Streptococcus rubneri* \| \| *Streptococcus salivarius* \| \| *Streptococcus sinensis* \| \| *Veillonella atypica* \| \| *Veillonella rogosae* \| |
|  |  |
| Only found in CD |  |
|  | \| *Abiotrophia defectiva* \| \| --- \| \| *Bacteroides cellulosilyticus* \| \| *Bacteroides coprocola* \| \| *Bifidobacterium merycicum* \| \| *Clostridium bolteae* \| \| *Eisenbergiella massiliensis* \| \| *Ilumatobacter nonamiensis* \| \| *Incertae Sedis* \| \| *Jonquetella anthropi* \| \| *Lactococcus chungangensis* \| \| *Lactococcus lactis* \| \| *Lactococcus piscium* \| \| *Lactococcus taiwanensis* \| \| *Megamonas funiformis* \| \| *Parabacteroides johnsonii* \| \| *Prevotella maculosa* \| \| *Streptobacillus moniliformis* \| \| *Streptococcus vestibularis* \| \| *Sutterella parvirubra* \| \| *Tannerella forsythia* \| |
| Only found in Control group |  |
|  | \| *Acidaminococcus intestini* \| \| --- \| \| *Adlercreutzia equolifaciens* \| \| *Alistipes finegoldii* \| \| *Alistipes obesi* \| \| *Alistipes putredinis* \| \| *Alistipes senegalensis* \| \| *Alistipes shahii* \| \| *Anaerofilum pentosovorans* \| \| *Anaeromassilibacillus senegalensis* \| \| *Anaerotaenia torta* \| \| *Bacteroides barnesiae* \| \| *Bacteroides caccae* \| \| *Bacteroides caecimuris* \| \| *Bacteroides eggerthii* \| \| *Bacteroides intestinalis* \| \| *Bacteroides oleiciplenus* \| \| *Bacteroides salyersiae* \| \| *Bacteroides stercorirosoris* \| \| *Barnesiella intestinihominis* \| \| *Butyricimonas faecihominis* \| \| *Butyricimonas paravirosa* \| \| *Butyricimonas synergistica* \| \| *Dialister propionicifaciens* \| \| *Eubacterium ventriosum* \| \| *Eubacterium xylanophilum* \| \| *Faecalicoccus acidiformans* \| \| *Holdemania filiformis* \| \| *Lachnospira pectinoschiza* \| \| *Massiliomicrobiota timonensis* \| \| *Murimonas intestini* \| \| *Negativibacillus massiliensis* \| \| *Oxalobacter formigenes* \| \| *Parabacteroides distasonis* \| \| *Parabacteroides gordonii* \| \| *Parabacteroides merdae* \| \| *Paraprevotella clara* \| \| *Phascolarctobacterium succinatutens* \| \| *Prevotella disiens* \| \| *Pseudobutyrivibrio ruminis* \| \| *Pseudoflavonifractor phocaeensis* \| \| *Ruminococcus albus* \| \| *Ruminococcus callidus* \| \| *Senegalimassilia anaerobia* \| \| *Succinatimonas hippei* \| \| *Sutterella massiliensis* \| \| *Sutterella wadsworthensis* \| \| *Sutturella timonensis* \| \| *Victivallis vadensis* \| |
|  |  |
| Found in all three groups |  |
|  | \| *Akkermansia muciniphila* \| \| --- \| \| *Alistipes onderdonkii* \| \| *Anaerostipes hadrus* \| \| *Bacteroides faecis* \| \| *Bacteroides fragilis* \| \| *Bacteroides massiliensis* \| \| *Bacteroides ovatus* \| \| *Bacteroides plebeius* \| \| *Bacteroides uniformis* \| \| *Bacteroides vulgatus* \| \| *Bacteroides xylanisolvens* \| \| *Bifidobacterium catenulatum* \| \| *Blautia obeum* \| \| *Butyricimonas virosa* \| \| *Collinsella aerofaciens* \| \| *Dialister succinatiphilus* \| \| *Dorea longicatena* \| \| *Escherichia coli* \| \| *Faecalibacterium prausnitzii* \| \| *Fusicatenibacter saccharivorans* \| \| *Gemmiger formicilis* \| \| *Kineothrix alysoides* \| \| *Lactobacillus rogosae* \| \| *Romboutsia timonensis* \| \| *Ruminococcus bromii* \| \| *Ruminococcus lactaris* \| \| *Ruminococcus torques* \| \| *Ruthenibacterium lactatiformans* \| \| *Streptococcus thermophilus* \| |
|  |  |
